# Chronic Pain After Spinal Cord Injury: Is There a Role for Neuron-Immune Dysregulation?

**DOI:** 10.3389/fphys.2020.00748

**Published:** 2020-07-07

**Authors:** Sílvia S. Chambel, Isaura Tavares, Célia D. Cruz

**Affiliations:** ^1^Department of Biomedicine, Experimental Biology Unit, Faculty of Medicine, University of Porto, Porto, Portugal; ^2^Translational NeuroUrology Group, Instituto de Investigação e Inovação em Saúde - i3S, Universidade do Porto, Porto, Portugal; ^3^Pain Research Group, Instituto de Investigação e Inovação em Saúde – i3S, Universidade do Porto, Porto, Portugal

**Keywords:** spinal cord injury, pain, glia, immune, astrocyte, microglia

## Abstract

Spinal cord injury (SCI) is a devastating event with a tremendous impact in the life of the affected individual and family. Traumatic injuries related to motor vehicle accidents, falls, sports, and violence are the most common causes. The majority of spinal lesions is incomplete and occurs at cervical levels of the cord, causing a disruption of several ascending and descending neuronal pathways. Additionally, many patients develop chronic pain and describe it as burning, stabbing, shooting, or shocking and often arising with no stimulus. Less frequently, people with SCI also experience pain out of context with the stimulus (e.g., light touch). While abolishment of the endogenous descending inhibitory circuits is a recognized cause for chronic pain, an increasing number of studies suggest that uncontrolled release of pro- and anti-inflammatory mediators by neurons, glial, and immune cells is also important in the emergence and maintenance of SCI-induced chronic pain. This constitutes the topic of the present mini-review, which will focus on the importance of neuro-immune dysregulation for pain after SCI.

## Introduction – Spinal Cord Injury

Spinal cord injury (SCI) causes major disturbances in sensory, motor, and autonomic function, leading to permanent loss of function and strongly impacting the physical, psychological, and social well-being of patients and caregivers (Braaf et al., 2017). It is estimated that 27 million people live worldwide with life-long consequences of SCI (James et al., 2019). Loss of function reflects the spinal level of the injury, with a high prevalence of injuries at the cervical and thoracic segments; the type of injury (compression, laceration, contusion, or ischemic insult); and the extent of the damage (Silva et al., 2014). Although life expectancy has greatly increased due to improved medical care, patients’ quality of life is severely compromised by several factors, including chronic pain (Yang et al., 2014; Kjell and Olson, 2016).

## Cellular Activation in SCI at the Lesion Site

Tissue remodeling begins immediately after SCI and continues for extended periods of time. SCI-induced alterations in the spinal cord occur in four steps (Rust and Kaiser, 2017). The (1) *primary injury* comprises the initial mechanical trauma (Rossignol et al., 2007). Within the first hours after injury, tissue damage initiates a series of destructive events that disrupt the spinal cord vasculature and blood-spinal cord barrier (BSCB). During *secondary injury* (2), platelets begin to invade the injury site and cause vasospasm, resulting in ischemia, glutamate release and oxidative stress (Park et al., 2004). The lesion extends and neuronal and glial cells undergo apoptosis and necrosis. Endogenous molecules, such as interleukin (IL)-1α, IL-33, ATP, and danger-associated molecular patterns (DAMPs) (Didangelos et al., 2016; Yang et al., 2017; Tran et al., 2018), are released by dying neurons and glial cells. This initiates a neuroinflammatory response, mediated by CNS astrocytes, microglia, and blood-borne neutrophils (Alizadeh et al., 2019).

*Astrocytes* are the first responders after a CNS injury (Pineau et al., 2010). Immediately after SCI, astrocytes become reactive (Xu et al., 1999; Bradbury and Burnside, 2019). Reactive astrocytes increase cytokine [e.g., IL-1β, IL-6, IL-12, tumor necrosis factor alpha (TNF-α) and interferon gamma (IFN-γ)] and chemokine (e.g., CCL2, CXCL1, and CXCL2) release in response to injury, which trigger recruitment of neutrophil and pro-inflammatory macrophages (Pineau et al., 2010; Alizadeh et al., 2019). Reactive astrocytes also release TGF-β and IL-10 that harness inflammation and avoid spreading of apoptotic and necrotic cells (Alizadeh et al., 2019) (Table 1).

**TABLE 1:** Innate and adaptive cells involved in the release of immune molecules at the injury site following spinal cord injury.

	**Type of cell**	**Function at the injury site**	**Pro-inflammatory molecules released at the injury site**	**Anti-inflammatory molecules released at the injury site**
*Innate immune cells*	Astrocytes	– Switch from quiescent to reactive following SCI – Recruit neutrophils and M1-macrophages – Involved in glial scar formation	IL-1β, IL-6, IL-12, TNF-α, IFN-γ, TGF-β CCL2, CXCL1, CXCL2	TGF-β, IL-10
	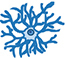			
	*Microglia*	– Clear cellular debris from neuronal and glial cell apoptosis – Aid in wound sealing	IL-1β, IL-2, IL-6, IL-12, IL-18, TNF-α, IFN-γ, C1q NO, ROS	TGF-β, IL-10, IGF-1
	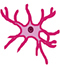			
	*Neutrophils*	– Attracted by CXCL1 and LTB4 – Clear debris from the injury site – Recruit monocytes/macrophages	IL-1β, IL-8, IL-12, TNF-α, IFN-γ MPO, MMP-9	Unknown
	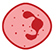			
	*Monocytes Macrophages*	– Polarization from M1-M2 subtypes	IL-1β, IL-6, IL-12, IL-18, TNF-α, IFN-γ NO, ROS	IL-10, TGF-β
	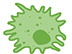			
*Adaptive immune cells*	*T-lymphocytes*	– Promote CNS fibrosis and autoimmunity	IL-1β, IL-12, TNF-α CCL2, CCL5, CXCL10	IL-2, IL-4, IL-5, IL-6, TGF-β
	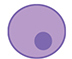			
	*B-lymphocytes*	– Promote autoimmunity and demyelination	Unknown	Unknown
	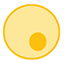			
				

*Neutrophils* are the first immune cells to respond to SCI, peaking at 24 h post-injury (Dusart and Schwab, 1994) but mostly disappear following the acute inflammatory phase (Neirinckx et al., 2014). Neutrophils are most likely attracted by CXCL1 and leukotriene-B4 (LTB4) secreted at the injury site. Invading neutrophils release pro-inflammatory molecules that exacerbate astrocytes and microglia activation at the lesion (Perkins and Tracey, 2000; Schomberg and Olson, 2012) (Table 1).

CNS resident *microglia* polarize within 5–15 min in response to injury, extending their processes toward the injury site. Once activated, microglia assume an amoeboid shape, proliferate and migrate to the lesion site (Popovich and Hickey, 2001; Sroga et al., 2003). There they play a crucial role in clearing cellular debris and aiding in wound sealing and glial scar maturation (Vilhardt, 2005; Loane and Byrnes, 2010). Microglia also express receptors to DAMPs released by injured neurons (Block et al., 2007; Loane and Byrnes, 2010). They are also responsible for releasing TNF-α, IL-1β, and C1q, which induce the activation of a subtype of astrocytes responsible for neuronal and oligodendrocyte cell death (Liddelow et al., 2017), an event linked to the emergence of neuropathic pain (Table 1).

While SCI-related microglial activation used to be perceived as an exclusively harmful event, it is now recognized that microglia also exert a neuroprotective role. Studies have shown cavity enlargement after early microglia ablation following SCI (Hines et al., 2009). More recently, using a *Cx3cr1*^creER^ mouse model, researchers demonstrated that eliminating microglia results in enhanced glial scar formation and neuronal and oligodendrocyte death, accompanied by poor locomotor performance (Bellver-Landete et al., 2019).

Twenty-four hours post-injury, circulating *monocytes* are recruited to the site of injury, where they differentiate into *macrophages*. Macrophages can be broadly divided into pro-inflammatory M1 or anti-inflammatory M2 macrophages. M1 macrophages are activated via toll-like receptors (TLRs) and IFN-γ, upregulating the expression of pro-inflammatory cytokines such as IL-6, IL-1β, IL-12, and TNF-α, and causing axonal retraction. Conversely, the M2 phenotype is activated by IL-13 or IL-4 (Gensel and Zhang, 2015). The shift from M1 to M2 macrophages is known to contribute to tissue healing (Deonarine et al., 2007; Nahrendorf et al., 2007) but does not occur, or is strongly impaired, in the lesioned spinal tissue (Kigerl et al., 2009). Kigerl et al. (2009) have demonstrated that, in a mouse model of midthoracic spinal contusion, M1 macrophage turnover is exacerbated in response to SCI, dominating the lesion site and nearby spared tissue, while M2 macrophages are short-lived, dissipating within 3–7 days post-SCI (Table 1).

Adaptive immune response also plays an important role in inflammatory response after SCI. After being activated in the spleen and bone marrow within 24 h post-SCI, *T- and B-lymphocytes* infiltrate the injured spinal cord, their numbers peaking at 7 days post-injury and remaining elevated in chronic stages of disease (Sroga et al., 2003; Jones, 2014). Activated T cells are particularly important on the modulation of inflammation following SCI as they can affect neuronal and glial survival via release of pro-inflammatory cytokines and chemokines (Jones, 2014), thus impairing recovery following SCI. Further information on the role of lymphocytes following SCI can be found elsewhere (Jones, 2014; Noble et al., 2018) (Table 1).

The third step comprises (3) *formation and maturation of the glial scar* by activated astrocytes. Recent studies revealed that microglia are also important players in glial scar formation, by accumulating around the scar and modulating astrocyte proliferation during scar formation via insulin-like growth factor 1 (IGF-1) (Bellver-Landete et al., 2019). Moreover, Zhou et al. (2020) have also demonstrated that microglia form a concentric physical barrier between the center of the lesion and its border, promoting wound compaction and recovery.

The final step in spinal cord remodeling after SCI consists of restricted (4) *structural tissue regeneration* occurring in the weeks and months after SCI (Shechter and Schwartz, 2013; Rust and Kaiser, 2017). However, the environment surrounding the scar is highly inhibitory axonal regrowth and reconnection. Thus, recovery of function is rarely achieved after a CNS injury, prompting the emergence of subsequent pathologies, including chronic neuropathic pain.

## Pain

Pain is defined by the International Association for the Study of Pain (IASP) as an unpleasant sensory and emotional experience, associated with actual or potential tissue damage, or described in terms of such damage (Loeser and Treede, 2008). While acute pain is usually well managed by patients and clinicians and resolved within a short period of time, chronic pain loses its protective role and becomes a disease in itself, even after resolving the triggering cause.

Pain perception is often initiated by the activation of nociceptors by noxious chemical, mechanical, or thermal stimuli in the periphery. These sensory neurons fall into two categories: medium-sized myelinated Aδ fibers and small-diameter, unmyelinated C-fibers. In the spinal cord, they synapse with relaying second-order neurons (either specific nociceptive neurons (NS) or wide-dynamic range neurons (WDR) or with inhibitory/excitatory spinal interneurons. Axons originated in these spinal cord neurons transmit ascending input to several supraspinal areas, namely the brainstem areas and the thalamus, the latter of which relays nociceptive information to cortical areas (Fenton et al., 2015; Boadas-Vaello et al., 2016). Nociceptive input is then processed and perceived, resulting in the activation of top-down descending modulation. These pathways may recruit higher brain centers, such as the prefrontal cortex and the amygdala, linked to cognitive and emotional aspects of pain. Top-down modulation also involves several supraspinal nuclei, including a midline relay circuit centered at the periaqueductal gray and rostral ventromedial medulla (PAG-RVM) (Heinricher et al., 2009; Boadas-Vaello et al., 2016). This key PAG-RVM circuit is connected with several brainstem regions, such as the locus coeruleus, the caudal ventrolateral medulla (VLM), and the dorsal reticular nucleus (DRt). The VLM and the DRt are reciprocally linked with the spinal cord, in circuits that may decrease or increase nociceptive information (Martins and Tavares, 2017). Descending pathways operate via release of serotonin, norepinephrine, and dopamine at supraspinal and spinal levels (Bourne et al., 2014). Considering the complexity of ascending and descending nociceptive neurotransmission, described in detail elsewhere (Boadas-Vaello et al., 2016; Martins and Tavares, 2017), it comes as no surprise that SCI strongly compromises these circuits and jeopardizes endogenous pain control circuits.

### Pain Following Spinal Cord Injury

Pain arising after SCI has life-long consequences, strongly impairing patients’ quality of life and often exceeding the impact of other functional disabilities. Pain manifests itself in several ways after SCI. While acute pain accompanies the injury and the recovery period, receding with tissue scarring, chronic pain emerges due to maladaptive neuroplasticity. More than 50% of SCI patients report chronic pain within a year of spinal lesion (Dijkers et al., 2009; Finnerup, 2013; Burke et al., 2017). Gabapentin, opioids and pregabalin remain gold standard for SCI-associated pain treatment, but are often ineffective and do not prevent pain worsening (Widerström-Noga, 2017).

Classically, SCI-related chronic pain can be divided into three major groups: nociceptive, neuropathic, or other/unknown pain (Bryce et al., 2012). Nociceptive SCI-derived pain includes musculoskeletal and visceral pain (Bryce et al., 2012; Shiao and Lee-Kubli, 2018). Neuropathic pain reflects SCI-induced damages in the somatosensory system and is divided into at-level, below-level, and above-level neuropathic pain. At-level pain usually emerges at early time points after SCI. It refers to pain felt in the dermatomes at the level of injury and includes central and peripheral components. Below-level pain is typically of central origin, felt diffusely below the level of injury and appearing when chronicity has set (Siddall et al., 2003; Finnerup, 2013). Finally, above-level neuropathic pain is now described as “other neuropathic pain” (Finnerup, 2013), relating to injury management, such as wheelchair pulling or pain following surgery.

### Neuropathic Pain Emergence Following Peripheral Nerve Injury

Spatial and temporal activation of glial cells in the spinal cord in several animal models of peripheral nerve injury has been vastly studied, but fewer data are available regarding SCI-induced neuropathic pain. This surely reflects the difficulties of reporting pain levels in animals with impaired mobility, as classical tests evaluate evoked responses to peripheral stimuli (Silva et al., 2014; Kramer et al., 2017). Therefore, much of our present knowledge on SCI-induced neuropathic pain stems from studies using models of peripheral nerve injury. Early studies in peripheral neuropathic pain reported increased GFAP immunostaining, an established marker for astrocytes, in the spinal dorsal horn (SDH) that correlated with emergence of hyperalgesia after sciatic nerve constriction injury (Garrison et al., 1991). Wagner and Myers demonstrated the role of TNF-α in hyperalgesia arising after sciatic nerve compression (Wagner and Myers, 1996), which has been proved to induce activity in primary nociceptors, hyperalgesia, and inflammation in rats (Sorkin et al., 1997; Junger and Sorkin, 2000), a process resulting from glial activation (Colburn et al., 1999; Zhuang et al., 2005). Macrophages are also involved in the emergence of neuropathic pain in models of peripheral nerve injury. Blockade of macrophage-colony stimulating factor signaling in a mouse model of partial sciatic nerve ligation prevented the development of injury-associated neuropathic pain (Lee et al., 2018). Further data on the role of neuroimmune interactions in peripheral nerve injury models can be found elsewhere (Malcangio, 2019; Tozaki-Saitoh and Tsuda, 2019).

### Neuropathic Pain Emergence Following Spinal Injury

#### The Role of Microglia and Astrocytes

Fewer studies have focused on the contribution of immune and glial cells to the emergence of neuropathic pain after SCI. Early studies by Peng et al. (2006) have demonstrated that a T13 unilateral hemisection produces bilateral microglia activation and TNF-α expression below the lesion level, correlating with hindpaw mechanical allodynia in SCI rats. Treating a T13 rat hemisection with etanercept, a TNF-α blocker, resulted in decreased mechanical allodynia and microglial activation. Treatment with minocycline, a microglial inhibitor, also improved pain-associated behaviors, demonstrating that TNF-α is critical in the establishment of neuropathic pain after SCI and dependent on microglial activation (Marchand et al., 2009). Thoracic spinal contusion also causes chronic activation of microglia as far as the lumbar spinal cord (Hains et al., 2003; Zai and Wrathall, 2005). When microglia activation is reversed by intrathecal administration of minocycline, lumbar SDH neurons hyperexcitability and pain-related behaviors decrease (Hains and Waxman, 2006; Tan et al., 2009). Early intrathecal administration of carbenoxolone, a gap junction decoupler, to T13 hemisected rats prevents astrocyte activation distant from the injury site and attenuates the development of thermal hyperalgesia and mechanical allodynia (Roh et al., 2010). Intrathecal administration of propentofylline (PPF), that prevents astrocytic and microglial activation and regulates the release of pro-inflammatory cytokines (Raghavendra et al., 2003; Arriagada et al., 2007), after a T13 transverse hemisection attenuates the development of mechanical allodynia and thermal hyperalgesia in the rat. Additionally, PPF reduces astrocyte and microglia activation away from the lesion site, thus decreasing hyperexcitability of lumbar WDR neurons and reducing below-level neuropathic pain in the rat (Gwak et al., 2008; Gwak and Hulsebosch, 2009).

At-level central neuropathic pain depends on p38-MAPK signaling. Following a thoracic contusion injury, p38α-MAPK is activated in neurons and microglia, but not in astrocytes, contributing to neuronal hyperexcitability (Crown et al., 2006, 2008; Gwak et al., 2009). Several other studies have identified microglial and astrocytic activation as key events for the development of below-level neuropathic pain (Detloff et al., 2008; Carlton et al., 2009; Gwak et al., 2012).

#### The Role of Immune Mediators

**FIGURE 1:**
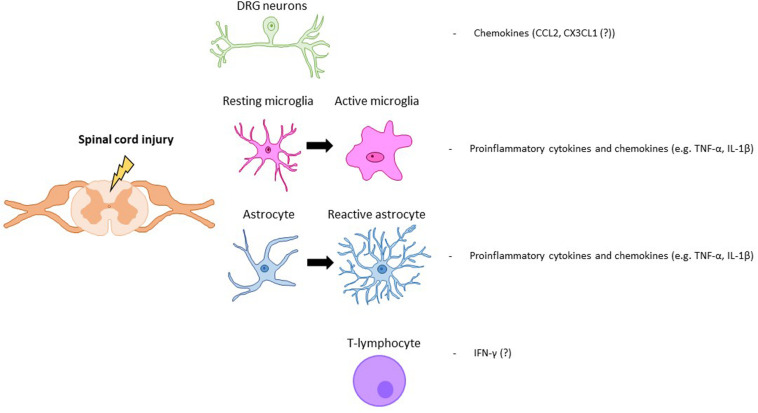
Key modulators of SCI pain. Neuronal, glial (astrocytes, microglia) and immune (T-lymphocyte) cells that are activated after a spinal cord injury. Key molecular mediators produced by those cells are also represented.

Following a SCI, interactions between nociceptive neurons, SDH neurons and glial cells are severely altered. After SCI, nociceptors become hyperactive and, upon stimulation, secrete increased amounts of glial modulators such as ATP, colony-stimulating factor-1 (CSF1), chemokines or caspase-6 (CASP6) (Ji et al., 2016). These molecules activate spinal microglia in the SDH, which respond by increasing the expression ATP and CX3CL1 receptors. Microglial cells increase secretion of TNF-α and IL-1β, responsible for enhancing excitatory and suppressing inhibitory synaptic transmissions in spinal cord lamina II neurons (Kawasaki et al., 2008). In the SDH, activated astrocytes are also able to potentiate excitatory synaptic transmission Nerve growth factor (NGF) release, which facilitates nociceptive neurotransmission, leads to neuropathic pain (Chen et al., 2014). In addition, NGF, which is also produced by infiltrating immune cells, may induce spinal expansion of the central terminals of nociceptors, amplifying synaptic input and further contributing to central neuropathic pain (Stroman et al., 2016; Gwak et al., 2017). SCI-induced dysregulation of neuron-glia crosstalk is not restricted to the spinal cord but has also been reported within dorsal root ganglia (DRG), affecting the interaction between Schwann cells within DRG neurons (Lee-Kubli et al., 2016) (Figure 1).

Chemokines are vital players in neuropathic pain development and maintenance following peripheral nerve injury (Zhang et al., 2017). They are likely involved in the mechanism of SCI-induced chronic neuropathic pain but the number of studies using SCI models to detail the contribution of specific chemokines is lacking.

#### The Role of Neurons

SCI-induced neuropathic pain also reflects neuronal hyperexcitability and increased spontaneous activity, observed both in DRG and spinal neurons, associated with behavioral responses to mechanical and thermal stimuli. This is concomitant with persistently activated microglia and astrocytes in SDH segments distant from the lesion site, the primary target of pro-inflammatory mediators released by those cells (Bedi et al., 2010). Therefore, there has been interest in developing strategies to control and reduce neuronal hyperexcitability, particularly at the spinal level. In a recent rodent SCI study, mouse cortical GABAergic interneurons, derived from the embryonic medial ganglionic eminence (MGE), have been transplanted into the spinal cord. This has resulted in a reduction in hyperexcitability associated with neuropathic pain (Braz et al., 2017). Efforts to translate these preclinical transplantation studies to the clinic are still ongoing.

## Clinical Trials on SCI-Associated Pain

Neuroimmune interactions on pain emergence after SCI are still rarely addressed on clinical trials. Kwon et al. (2009) found increased levels of TNF-α receptor 1 in cerebrospinal fluid (CSF), which correlated with the emergence of neuropathic pain. A phase II trial on the effects of minocycline administration after traumatic SCI revealed, at 1-year follow-up, improved motor recovery but effects on pain improvement were not fully satisfactory (Casha et al., 2012; Badhiwala et al., 2018b).

## Discussion and Conclusion

Alleviation of neuropathic pain arising after SCI is still an unmet need for most SCI patients. Current pharmacological pain treatments are unsatisfactory and there is an urgent need to develop more effective strategies. Therefore, neuroimmune dysregulation in the context of CNS injuries has emerged as a putative target for improved treatment of SCI-induced neuropathic pain. Accumulating evidence supports a role for neuro-immune dysregulation, often reflecting altered crosstalk between neurons, activated glial cells and invading immune cells (macrophages and T-cells). Dysregulation occurs as a consequence of the inflammatory response to SCI. This response is necessary in the early post-SCI stages to close the injury site but the resulting scar prevents full tissue regeneration and leads to maladaptive neuroplasticity and chronic pain. The challenge is, therefore, to control the inflammatory response and promote tissue repair, both at a structural and functional levels. Presently, methylprednisolone, a potent anti-inflammatory drug, is the only medication in clinical practice used to treat SCI in early stages. Methylprednisolone has a widespread activity and is able to reduce cytokine release (Ulndreaj et al., 2016) but reported side effects, and lack of positive outcomes in pain control, among other chronic problems, has led investigators to question its use (Evaniew et al., 2016; Monteiro et al., 2018). The identification of glial cells as important sources of pro- and anti-inflammatory neuromodulators allowed the identification of carbenoxolone, minocycline, and PPF as eventual therapeutic tools (Shultz and Zhong, 2017; Badhiwala et al., 2018a). Although these drugs have produced interesting results in pre-clinical and early clinical trials, dosages and timing for intervention need to be critically defined in the future for effective pain management.

## Author Contributions

SC wrote the first version of the manuscript and edited the subsequent versions. IT and CC corrected and edited the original manuscript and subsequent versions. All authors contributed to the article and approved the submitted version.

## Conflict of Interest

The authors declare that the research was conducted in the absence of any commercial or financial relationships that could be construed as a potential conflict of interest.
